# Transactions of the American Medical Association

**Published:** 1849-01

**Authors:** 


*Transactions of the American Medical Association.* Instituted 1847 Vol. I, pp. 463. Philadelphia, printed for the Association, by T. K. & P. G. Collins, 1848.

Through the politeness of J. B. Herrick, M. D., Delegate of Rush Medical College to the last meeting of the Association, we have been favored with a copy of the above work. It contains first the proceedings of the last meeting of this body, a synopsis of which was given in a former number of this Journal. This makes 46 pages—the remaining 359 pages are made up of the reports of commitees, which form highly interesting documents upon the several subjects which had been referred to them by the association.

The work can be had of the publishing committee, through their chairman, Dr. Isaac Hays, of Philadelphia. E.

